# Cost impact of hospital acquired diagnoses and impacts for funding based on quality signals

**DOI:** 10.1186/1472-6963-15-S2-A7

**Published:** 2015-06-15

**Authors:** Jim Pearse, Deniza Mazevska, Terri Jurgens Jackson

**Affiliations:** 1 Health Policy Analysis, St Leonards, New South Wales, 2065, Australia; 2 Australian Health Services Research Institute, University of Wollongong, Wollongong, New South Wales, 2522, Australia; 3 Northern Clinical Research Centre, University of Melbourne, Epping, Victoria, 3076, Australia

## Background

Internationally, there have been efforts to adjust hospital funding based on the quality of care provided by the hospital. A variety of approaches has been used by different countries and payers. Incorporating quality signals into activity-based funding is also a possibility for Australia.

This study set out to explore the cost impact of potentially poor quality care in Australian hospitals, and to understand the implications from a funding perspective.

## Materials and methods

Since 2008, Australia has incorporated into its routinely collected hospital data a flag to indicate whether each diagnosis was pre-existing at the time of admission, or if it arose during the hospital stay (i.e., a hospital-acquired condition). This is known as the Condition Onset Flag (COF).

This study used the Admitted Patient Care National Minimum Data Set (NMDS) and the National Hospital Cost Data Collection for 2011-2012. These are routine national collections of admitted patient data, and activity-based costing data, respectively.

The analysis of the cost impact was limited to medium and large hospitals (i.e. facilities with approximately 2,000 or more separations annually, using the national peer group classification). Within these peer groups, the analysis included only hospitals that coded the COF. The analysis was further restricted to acute episodes of admitted patient care (for example, excluding rehabilitation and palliative care episodes), and a set of Australian Refined Diagnostic Related Groups (AR-DRGs) identified by the Australian Commission on Safety and Quality in Health Care (based on volume, cost, and priority areas for quality and safety initiatives). Just over 400,000 episodes were included in the detailed analysis.

The primary analysis was based on a Generalized Linear Model in which differences in cost or length of stay were modeled using the presence of hospital acquired conditions as explanatory variables, together with a range of control variables (including age, emergency care status, and the presence of other comorbidities). Alternative specifications for the presence of hospital-acquired conditions were estimated, using the Classification of Hospital Acquired Conditions (CHADx) [1]. Models were estimates for each adjacent AR-DRG.

## Results

An estimate was made of the total incremental impact of the presence of hospital-acquired conditions, both within the sample, and scaling it to reflect all acute episodes allocated to the selected conditions and/or interventions (mapped to Adjacent DRGs) in the selected public and private hospitals.

Across the sample of conditions and/or interventions identified by the Commission, the mean incremental impact of the presence of any COF diagnosis was estimated to be 9,244 AUD (with a median of 6,710 AUD).

Scaled to all acute episodes, hospital-acquired conditions accounted for between 12% and 16.5% of total costs within the sample, and between 11.6% and 15.9% of costs across all hospitals for the selected conditions. Across all acute episodes assigned to Adjacent DRGs of the selected conditions, the incremental cost of hospital-acquired conditions was estimated to be between 634 million and 896 million AUD. To place this estimate in context, total expenditures for public hospitals were 40,384 million AUD in 2011-2012, of which approximately 28,000 million AUD (70%) was related to admitted patients.

The highest costs were associated with less costly (per case) but more frequent complications. Total cost impacts of these conditions ranged from 10.9 million AUD for pressure ulcers (1,866 episodes) to 27.4 million AUD for electrolyte disorders without dehydration (9,808 episodes).

## Conclusions

This study’s estimate of the cost impact of hospital acquired diagnoses offers insights into costs that could be shifted, if incorporating quality signals into activity-based funding reduced these complications.

Four broad options for how quality signals might be incorporated into funding are:

1. Maintain the current core activity-based funding approach and create a separate funding / payment stream related to performance against quality-related measures / benchmarks, including those based on analysis of hospital acquired conditions.

2. Exclude all hospital-acquired complications when assigning DRGs, so the presence of such a diagnosis does not impact the patient’s DRG (and the facilities’ subsequent funding).

3. Exclude a subset of hospital-acquired complications in the AR-DRG assignment.

4. Exclude the costs of hospital-acquired complications in calculating the price weights for each DRG.

While not all hospital-acquired conditions can be prevented with current medical knowledge, incorporating quality signals into pricing might motivate greater efforts to reduce them, where possible.

The limitations of the study should be noted. We found coding of the COF to vary between hospitals and states. The study is vulnerable to endogeneity bias because of the circular relationship between length of stay and rates of hospital-acquired harms. Length of stay may be extended by complications that arise during the admission, but longer stays also expose patients to a higher probability that a hospital-acquired condition will occur. Both are highly correlated with higher costs.
